# (*meso*-5,5,7,12,12,14-Hexamethyl-1,4,8,11-tetra­aza­cyclo­tetra­deca­ne)copper(II) bis­[*O*,*O*′-(*o*-phenyl­ene)dithio­phosphate]

**DOI:** 10.1107/S1600536810046684

**Published:** 2010-11-17

**Authors:** Jian-Shen Feng, Li-Ke Zou, Bin Xie, Yang-Guang Xiang, Chuan Lai

**Affiliations:** a College of Chemistry and Pharmaceutical Engineering, Sichuan University of Science and Engineering , 643000 Zigong, Sichuan, People’s Republic of China

## Abstract

In the title compound, [Cu(C_16_H_36_N_4_)](C_6_H_4_O_2_PS_2_)_2_, the Cu^II^ cation lies on an inversion center and is chelated by the macrocyclic tetra­amine ligand in a slightly distorted CuN_4_ square-planar geometry. The axial positions are occupied by two *O*,*O*′-(*o*-phenyl­ene)dithio­phosphate anions with long Cu⋯S distances of 3.0940 (7) Å. Inter­molecular N—H⋯S and C—H⋯O hydrogen bonding is present between the anions and the cation and helps to stabilize the crystal structure.

## Related literature

For applications of macrocyclic tetra­amine compounds, see: Groeta *et al.* (2000 ▶); Aoki & Kimura (2002 ▶). For related structures, see: Feng *et al.* (2009 ▶); He *et al.* (2010 ▶); Xie *et al.* (2009 ▶).
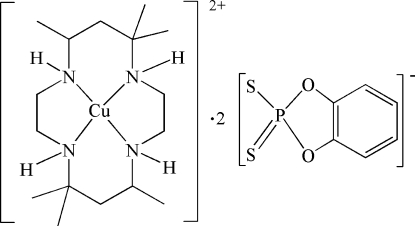


## Experimental

### 

#### Crystal data

[Cu(C_16_H_36_N_4_)](C_6_H_4_O_2_PS_2_)_2_
*M* *_r_* = 754.39
Monoclinic, 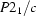

*a* = 12.3107 (4) Å
*b* = 12.1612 (3) Å
*c* = 12.3703 (4) Å
β = 107.136 (3)°
*V* = 1769.78 (9) Å^3^
*Z* = 2
Mo *K*α radiation
μ = 0.98 mm^−1^
*T* = 294 K
0.38 × 0.34 × 0.28 mm

#### Data collection

Oxford Diffraction Xcalibur Eos diffractometer
Absorption correction: multi-scan (*CrysAlis PRO RED*; Oxford Diffraction, 2009 ▶) *T*_min_ = 0.697, *T*_max_ = 0.761
7203 measured reflections
3621 independent reflections
2662 reflections with *I* > 2σ(*I*)
*R*_int_ = 0.018

#### Refinement

*R*[*F* ^2^ > 2σ(*F* ^2^)] = 0.029
*wR*(*F* ^2^) = 0.072
*S* = 1.02
3621 reflections
199 parameters
H-atom parameters constrained
Δρ_max_ = 0.23 e Å^−3^
Δρ_min_ = −0.37 e Å^−3^

### 

Data collection: *CrysAlis PRO CCD* (Oxford Diffraction, 2009 ▶); cell refinement: *CrysAlis PRO CCD*; data reduction: *CrysAlis PRO RED* (Oxford Diffraction, 2009 ▶); program(s) used to solve structure: *SHELXS97* (Sheldrick, 2008 ▶); program(s) used to refine structure: *SHELXL97* (Sheldrick, 2008 ▶); molecular graphics: *ORTEP-3 for Windows* (Farrugia, 1997 ▶); software used to prepare material for publication: *SHELXL97*.

## Supplementary Material

Crystal structure: contains datablocks I, global. DOI: 10.1107/S1600536810046684/xu5086sup1.cif

Structure factors: contains datablocks I. DOI: 10.1107/S1600536810046684/xu5086Isup2.hkl

Additional supplementary materials:  crystallographic information; 3D view; checkCIF report

## Figures and Tables

**Table 1:** Hydrogen-bond geometry (Å, °)

*D*—H⋯*A*	*D*—H	H⋯*A*	*D*⋯*A*	*D*—H⋯*A*
N1—H1⋯S2	0.91	2.62	3.4849 (16)	160
N2—H2⋯S1^i^	0.91	2.60	3.2715 (16)	132
C1—H1*A*⋯O1^ii^	0.97	2.52	3.449 (2)	160

Symmetry codes: (i) 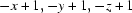; (ii) 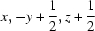.

**Fig. 1.**
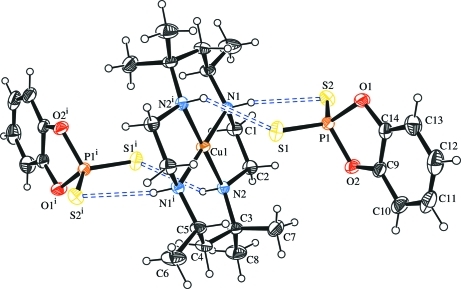
The molecular structure of compound, showing the atom-numbering scheme. Displacement ellipsoids are drawn at the 30% probability level. H atoms are represented as small spheres of arbitrary radii. Hydrogen-bonds are shown as dashed lines [symmetry code: (i) -x + 1, -y + 1, -z + 1].

## supplementary crystallographic information

### Comment

The synthesis and structural investigation of tetraamine macrocycle have attracted much attention due to their wide potential applications (Groeta *et al.*, 2000; Aoki & Kimura, 2002). In our quest for mimetic hydrolases, we have recently reported several structures of tetramine macrocyclic transition metal adducts with O,O'-dialkyldithiophosphate (Feng *et al.*, 2009; Xie *et al.*, 2009; He *et al.*, 2010). Herein, we report the structure of [Cu(*meso*-hmta)] [(*o*-C_6_H_4_O_2_)PS_2_]_2_, where *meso*-hmta is *meso*-5,5,7,12,12,14-hexamethyl-1,4,8,11-tetraazacyclotetradecane.

The molecular of the title adduct comprises a complex mononuclear [Cu(*meso*-hmta)]^2+^ cation and two *O*,*O*'-(1,2-phenylene)dithiophosphate anions. Its structure is remarkably similar to the analogues, [Cu(*trans*-[14]dien)][S_2_P(OC_6_H_4_Me-4)_2_]_2_ (He *et al.*, 2010), where *trans*-[14]dien is *meso*-5,7,7,12,14,14-hexamethyl-1,4,8,11-tetraazacyclotetradeca -4,11-diene. The Cu^II^ atom lies on an inversion center and is chelated by tetraamine macrocycle ligand with a relatively undistorted CuN_4_ square-planar geometry (Fig.1). Two uncoordinated *O*,*O*'- (1,2-phenylene)dithiophosphate anions occupy at the axial positions with the longer Cu···S distances of 3.0940 (7) Å, forming an octahedral asymmetric unit. Intermolecular N—H···S hydrogen bonding is present between the anions and the cation and help to stabilize the crystal structure (Table 1). The strain in the *O*,*O*'- (1,2-phenylene)dithiophosphate anions is illustrated by the distorted tetrahedral angles of P atoms, which range between 94.43 (7) and 120.01 (4)°.

### Experimental

[Et_3_NH][(o-C_6_H_4_O_2_)PS_2_] was prepared by adding P_2_S_5_ (0.05 mol, 11.1 g) and catechol (0.1 mol, 11 g) to a solution of triethylamine (15 mL) in 50 mL toluene with stirring. The mixture was stirred for 45 min at 368 K and then refluxed for 15 min. After cooled to room temperature, the precipitate was filtered off and washed successively with methanol, diethyl ether and acetone. The white crystalline product was obtained by recrystallization in hot methanol, yield 79%.

A solution of *meso*-5,5,7,12,12,14- hexamethyl-1,4,8,11- tetraazacyclotetradecane dihydrate (0.32 g, 1 mmol) and CuCl_2_.2H_2_O (0.17 g, 1 mmol) in 20 ml methanol was quickly added to [Et_3_NH][(o-C_6_H_4_O_2_)PS_2_] (2 mmol, 0.61 g) dissolved in 20 ml hot methanol with stirring. The mixture was refluxed for 4 h and cooled to room temperature, the precipitate was filtered off, washed with diethyl ether and recrystallized from benzene to leave a dark-violet solid, which was dissolved in hot methanol and filtered. The filtrate was kept at room temperature and dark-violet block crystals of the title compound suitable for X-ray diffraction studies were obtained after a week.

### Refinement

H atoms on C and N atoms were fixed geometrically and treated as riding, with C—H = 0.98 (methine), 0.97 (methylene), 0.96 (methyl), 0.93 (aromatic) and N—H = 0.91 Å. The *U*_iso_(H) = 1.5*U*_eq_(C) for methyl and *U*_iso_(H) = 1.2*U*_eq_(C,N) for the other H atoms.

### Crystal data

**Table:**

[Cu(C_16_H_36_N_4_)](C_6_H_4_O_2_PS_2_)_2_	*F*(000) = 790
*M**_r_* = 754.39	*D*_x_ = 1.416 Mg m^−^^3^
Monoclinic, *P*2_1_/*c*	Mo *K*α radiation, λ = 0.7107 Å
Hall symbol: -P 2ybc	Cell parameters from 4041 reflections
*a* = 12.3107 (4) Å	θ = 3.2–29.2°
*b* = 12.1612 (3) Å	µ = 0.98 mm^−^^1^
*c* = 12.3703 (4) Å	*T* = 294 K
β = 107.136 (3)°	Block, dark-violet
*V* = 1769.78 (9) Å^3^	0.38 × 0.34 × 0.28 mm
*Z* = 2	

### Data collection

**Table:**

Oxford Diffraction Xcalibur Eos diffractometer	3621 independent reflections
Radiation source: fine-focus sealed tube	2662 reflections with *I* > 2σ(*I*)
graphite	*R*_int_ = 0.018
Detector resolution: 16.0874 pixels mm^-1^	θ_max_ = 26.4°, θ_min_ = 3.3°
ω scans	*h* = −14→15
Absorption correction: multi-scan (*CrysAlis PRO* RED; Oxford Diffraction, 2009)	*k* = −15→14
*T*_min_ = 0.697, *T*_max_ = 0.761	*l* = −15→8
7203 measured reflections	

### Refinement

**Table:**

Refinement on *F*^2^	Primary atom site location: structure-invariant direct methods
Least-squares matrix: full	Secondary atom site location: difference Fourier map
*R*[*F*^2^ > 2σ(*F*^2^)] = 0.029	Hydrogen site location: inferred from neighbouring sites
*wR*(*F*^2^) = 0.072	H-atom parameters constrained
*S* = 1.02	*w* = 1/[σ^2^(*F*_o_^2^) + (0.0358*P*)^2^] where *P* = (*F*_o_^2^ + 2*F*_c_^2^)/3
3621 reflections	(Δ/σ)_max_ = 0.001
199 parameters	Δρ_max_ = 0.23 e Å^−^^3^
0 restraints	Δρ_min_ = −0.37 e Å^−^^3^

### Special details

**Table:**

Geometry. All e.s.d.'s (except the e.s.d. in the dihedral angle between two l.s. planes) are estimated using the full covariance matrix. The cell e.s.d.'s are taken into account individually in the estimation of e.s.d.'s in distances, angles and torsion angles; correlations between e.s.d.'s in cell parameters are only used when they are defined by crystal symmetry. An approximate (isotropic) treatment of cell e.s.d.'s is used for estimating e.s.d.'s involving l.s. planes.
Refinement. Refinement of *F*^2^ against ALL reflections. The weighted *R*-factor *wR* and goodness of fit *S* are based on *F*^2^, conventional *R*-factors *R* are based on *F*, with *F* set to zero for negative *F*^2^. The threshold expression of *F*^2^ > σ(*F*^2^) is used only for calculating *R*-factors(gt) *etc*. and is not relevant to the choice of reflections for refinement. *R*-factors based on *F*^2^ are statistically about twice as large as those based on *F*, and *R*- factors based on ALL data will be even larger.

### Fractional atomic coordinates and isotropic or equivalent isotropic displacement parameters (Å^2^)

**Table:**

	*x*	*y*	*z*	*U*_iso_*/*U*_eq_	
C1	0.60607 (18)	0.32263 (15)	0.63651 (17)	0.0421 (5)	
H1A	0.6460	0.2530	0.6438	0.050*	
H1B	0.5573	0.3210	0.6854	0.050*	
C6	0.5147 (2)	0.85983 (17)	0.5043 (2)	0.0750 (9)	
H6B	0.4588	0.8709	0.5434	0.113*	
H6A	0.4810	0.8734	0.4250	0.113*	
H6C	0.5770	0.9096	0.5336	0.113*	
C3	0.69922 (17)	0.62275 (15)	0.67689 (17)	0.0385 (5)	
C4	0.62020 (18)	0.72195 (15)	0.64523 (18)	0.0448 (5)	
H4A	0.5636	0.7154	0.6853	0.054*	
H4B	0.6647	0.7872	0.6739	0.054*	
C2	0.69001 (16)	0.41537 (15)	0.67066 (18)	0.0417 (5)	
H2B	0.7315	0.4094	0.7503	0.050*	
H2A	0.7441	0.4122	0.6275	0.050*	
C5	0.55801 (18)	0.74158 (14)	0.52163 (18)	0.0412 (5)	
H5	0.6116	0.7305	0.4777	0.049*	
C8	0.7587 (2)	0.62675 (19)	0.80430 (19)	0.0669 (7)	
H8A	0.7031	0.6214	0.8445	0.100*	
H8C	0.7993	0.6949	0.8231	0.100*	
H8B	0.8111	0.5665	0.8253	0.100*	
C7	0.78770 (19)	0.62258 (17)	0.6133 (2)	0.0589 (7)	
H7B	0.8442	0.5677	0.6449	0.088*	
H7C	0.8232	0.6936	0.6198	0.088*	
H7A	0.7516	0.6063	0.5349	0.088*	
P1	0.76165 (4)	0.41284 (4)	0.34491 (4)	0.03621 (14)	
S1	0.62560 (5)	0.50447 (5)	0.31713 (5)	0.05140 (17)	
S2	0.77079 (5)	0.27519 (4)	0.42553 (5)	0.04891 (16)	
O1	0.79072 (12)	0.39508 (11)	0.22398 (12)	0.0475 (4)	
O2	0.87362 (11)	0.49007 (10)	0.40083 (12)	0.0452 (4)	
C9	0.91021 (16)	0.53308 (17)	0.31328 (19)	0.0411 (5)	
N1	0.53721 (13)	0.33932 (11)	0.51843 (12)	0.0309 (4)	
H1	0.5849	0.3263	0.4759	0.037*	
C10	0.98455 (17)	0.61848 (17)	0.3208 (2)	0.0554 (6)	
H10	1.0162	0.6554	0.3886	0.067*	
C13	0.8889 (2)	0.50583 (19)	0.1159 (2)	0.0611 (7)	
H13	0.8572	0.4685	0.0483	0.073*	
C14	0.86346 (18)	0.47825 (17)	0.21245 (19)	0.0438 (5)	
Cu1	0.5000	0.5000	0.5000	0.03160 (11)	
N2	0.62678 (12)	0.52077 (11)	0.64861 (13)	0.0315 (4)	
H2	0.5886	0.5211	0.7014	0.038*	
C11	1.0108 (2)	0.6478 (2)	0.2226 (3)	0.0656 (8)	
H11	1.0605	0.7058	0.2246	0.079*	
C12	0.9647 (2)	0.5925 (2)	0.1231 (3)	0.0713 (8)	
H12	0.9844	0.6133	0.0590	0.086*	

### Atomic displacement parameters (Å^2^)

**Table:**

	*U*^11^	*U*^22^	*U*^33^	*U*^12^	*U*^13^	*U*^23^
C1	0.0483 (13)	0.0289 (10)	0.0428 (12)	0.0079 (9)	0.0039 (10)	0.0100 (9)
C6	0.095 (2)	0.0260 (11)	0.086 (2)	−0.0022 (12)	−0.0002 (17)	0.0049 (12)
C3	0.0387 (11)	0.0351 (11)	0.0366 (12)	−0.0048 (9)	0.0034 (9)	−0.0070 (9)
C4	0.0538 (14)	0.0319 (11)	0.0451 (13)	−0.0057 (10)	0.0093 (11)	−0.0101 (10)
C2	0.0377 (11)	0.0350 (11)	0.0446 (13)	0.0084 (10)	−0.0001 (10)	0.0061 (9)
C5	0.0477 (13)	0.0242 (10)	0.0493 (14)	−0.0043 (9)	0.0107 (11)	0.0009 (9)
C8	0.0761 (18)	0.0619 (15)	0.0461 (15)	−0.0064 (14)	−0.0075 (13)	−0.0119 (12)
C7	0.0427 (13)	0.0543 (14)	0.0793 (19)	−0.0091 (11)	0.0173 (13)	−0.0009 (13)
P1	0.0333 (3)	0.0453 (3)	0.0317 (3)	−0.0046 (2)	0.0121 (2)	−0.0077 (2)
S1	0.0385 (3)	0.0599 (4)	0.0593 (4)	0.0075 (3)	0.0198 (3)	0.0164 (3)
S2	0.0556 (4)	0.0443 (3)	0.0498 (4)	0.0056 (3)	0.0203 (3)	−0.0009 (3)
O1	0.0522 (9)	0.0577 (9)	0.0393 (9)	−0.0202 (8)	0.0239 (7)	−0.0174 (7)
O2	0.0381 (8)	0.0580 (9)	0.0381 (8)	−0.0114 (7)	0.0092 (7)	−0.0122 (7)
C9	0.0276 (11)	0.0434 (12)	0.0522 (14)	−0.0006 (9)	0.0116 (10)	0.0005 (10)
N1	0.0352 (8)	0.0224 (8)	0.0354 (9)	0.0057 (7)	0.0107 (7)	0.0047 (7)
C10	0.0321 (12)	0.0496 (13)	0.0775 (19)	−0.0012 (10)	0.0050 (12)	0.0001 (13)
C13	0.0662 (16)	0.0712 (17)	0.0568 (16)	−0.0033 (14)	0.0352 (14)	−0.0009 (13)
C14	0.0371 (12)	0.0526 (14)	0.0468 (14)	−0.0015 (10)	0.0201 (10)	−0.0047 (10)
Cu1	0.03122 (18)	0.02070 (16)	0.0368 (2)	0.00205 (14)	0.00057 (14)	0.00432 (14)
N2	0.0308 (9)	0.0325 (9)	0.0305 (9)	0.0028 (7)	0.0080 (7)	0.0025 (7)
C11	0.0361 (13)	0.0555 (15)	0.109 (2)	0.0023 (12)	0.0270 (15)	0.0229 (16)
C12	0.0622 (17)	0.0794 (19)	0.087 (2)	0.0072 (15)	0.0440 (17)	0.0226 (16)

### Geometric parameters (Å, °)

**Table:**

C1—H1A	0.9700	C7—H7A	0.9600
C1—H1B	0.9700	P1—S1	1.9560 (7)
C1—C2	1.504 (3)	P1—S2	1.9348 (8)
C1—N1	1.471 (2)	P1—O1	1.6517 (14)
C6—H6B	0.9600	P1—O2	1.6429 (14)
C6—H6A	0.9600	O1—C14	1.386 (2)
C6—H6C	0.9600	O2—C9	1.392 (2)
C6—C5	1.527 (3)	C9—C10	1.369 (3)
C3—C4	1.527 (3)	C9—C14	1.382 (3)
C3—C8	1.531 (3)	N1—C5^i^	1.499 (2)
C3—C7	1.520 (3)	N1—H1	0.9100
C3—N2	1.507 (2)	N1—Cu1	2.0047 (13)
C4—H4A	0.9700	C10—H10	0.9300
C4—H4B	0.9700	C10—C11	1.391 (3)
C4—C5	1.514 (3)	C13—H13	0.9300
C2—H2B	0.9700	C13—C14	1.363 (3)
C2—H2A	0.9700	C13—C12	1.393 (3)
C2—N2	1.483 (2)	Cu1—N1^i^	2.0047 (13)
C5—H5	0.9800	Cu1—N2	2.0466 (15)
C5—N1^i^	1.498 (2)	Cu1—N2^i^	2.0466 (15)
C8—H8A	0.9600	N2—H2	0.9100
C8—H8C	0.9600	C11—H11	0.9300
C8—H8B	0.9600	C11—C12	1.370 (4)
C7—H7B	0.9600	C12—H12	0.9300
C7—H7C	0.9600		
			
C1—C2—H2B	110.0	S2—P1—S1	120.01 (4)
C1—C2—H2A	110.0	O1—P1—S1	108.55 (6)
C1—N1—C5^i^	113.81 (14)	O1—P1—S2	111.01 (6)
C1—N1—H1	105.6	O2—P1—S1	108.48 (5)
C1—N1—Cu1	107.04 (11)	O2—P1—S2	111.31 (6)
H1A—C1—H1B	108.3	O2—P1—O1	94.43 (7)
C6—C5—H5	108.4	C9—O2—P1	108.12 (12)
H6B—C6—H6A	109.5	C9—C10—H10	121.4
H6B—C6—H6C	109.5	C9—C10—C11	117.2 (2)
H6A—C6—H6C	109.5	C9—C14—O1	111.67 (19)
C3—C4—H4A	107.7	N1—C1—H1A	109.9
C3—C4—H4B	107.7	N1—C1—H1B	109.9
C3—C8—H8A	109.5	N1—C1—C2	108.83 (15)
C3—C8—H8C	109.5	N1^i^—C5—C6	111.41 (17)
C3—C8—H8B	109.5	N1^i^—C5—C4	110.13 (15)
C3—C7—H7B	109.5	N1^i^—C5—H5	108.4
C3—C7—H7C	109.5	N1—Cu1—N1^i^	180.0
C3—C7—H7A	109.5	N1^i^—Cu1—N2^i^	85.98 (6)
C3—N2—Cu1	123.54 (11)	N1—Cu1—N2^i^	94.02 (6)
C3—N2—H2	103.1	N1—Cu1—N2	85.98 (6)
C4—C3—C8	108.33 (17)	N1^i^—Cu1—N2	94.02 (6)
C4—C5—C6	110.00 (17)	C10—C9—O2	126.6 (2)
C4—C5—H5	108.4	C10—C9—C14	121.3 (2)
H4A—C4—H4B	107.1	C10—C11—H11	119.4
C2—C1—H1A	109.9	C13—C14—O1	126.3 (2)
C2—C1—H1B	109.9	C13—C14—C9	122.0 (2)
C2—N2—C3	115.21 (15)	C13—C12—H12	119.3
C2—N2—Cu1	106.18 (11)	C14—O1—P1	108.47 (12)
C2—N2—H2	103.1	C14—C9—O2	112.15 (18)
H2B—C2—H2A	108.4	C14—C13—H13	121.5
C5—C6—H6B	109.5	C14—C13—C12	116.9 (2)
C5—C6—H6A	109.5	Cu1—N1—H1	105.6
C5—C6—H6C	109.5	Cu1—N2—H2	103.1
C5—C4—C3	118.56 (17)	N2—C3—C4	107.57 (15)
C5—C4—H4A	107.7	N2—C3—C8	109.63 (16)
C5—C4—H4B	107.7	N2—C3—C7	110.12 (16)
C5^i^—N1—H1	105.6	N2—C2—C1	108.46 (15)
C5^i^—N1—Cu1	118.17 (12)	N2—C2—H2B	110.0
H8A—C8—H8C	109.5	N2—C2—H2A	110.0
H8A—C8—H8B	109.5	N2—Cu1—N2^i^	180.0
H8C—C8—H8B	109.5	C11—C10—H10	121.4
C7—C3—C4	111.59 (18)	C11—C12—C13	121.4 (3)
C7—C3—C8	109.55 (19)	C11—C12—H12	119.3
H7B—C7—H7C	109.5	C12—C13—H13	121.5
H7B—C7—H7A	109.5	C12—C11—C10	121.1 (2)
H7C—C7—H7A	109.5	C12—C11—H11	119.4
			
C1—C2—N2—C3	−179.14 (16)	S1—P1—O2—C9	−90.46 (12)
C1—C2—N2—Cu1	−38.57 (18)	S2—P1—O1—C14	−135.33 (12)
C1—N1—Cu1—N2	15.68 (13)	S2—P1—O2—C9	135.39 (11)
C1—N1—Cu1—N2^i^	−164.32 (13)	O1—P1—O2—C9	20.78 (13)
C3—C4—C5—C6	−162.4 (2)	O2—P1—O1—C14	−20.48 (14)
C3—C4—C5—N1^i^	74.4 (2)	O2—C9—C10—C11	179.29 (18)
C4—C3—N2—C2	178.22 (16)	O2—C9—C14—O1	0.8 (3)
C4—C3—N2—Cu1	45.3 (2)	O2—C9—C14—C13	−179.1 (2)
C2—C1—N1—C5^i^	−173.86 (16)	C9—C10—C11—C12	−0.6 (3)
C2—C1—N1—Cu1	−41.42 (18)	N1—C1—C2—N2	54.5 (2)
C5^i^—N1—Cu1—N2	145.70 (14)	N1—Cu1—N2—C3	149.25 (14)
C5^i^—N1—Cu1—N2^i^	−34.30 (14)	N1^i^—Cu1—N2—C3	−30.75 (14)
C8—C3—C4—C5	175.34 (19)	N1^i^—Cu1—N2—C2	−167.17 (12)
C8—C3—N2—C2	−64.2 (2)	N1—Cu1—N2—C2	12.83 (12)
C8—C3—N2—Cu1	162.85 (14)	C10—C9—C14—O1	−179.94 (17)
C7—C3—C4—C5	54.7 (2)	C10—C9—C14—C13	0.2 (3)
C7—C3—N2—C2	56.4 (2)	C10—C11—C12—C13	0.7 (4)
C7—C3—N2—Cu1	−76.55 (19)	C14—C9—C10—C11	0.1 (3)
P1—O1—C14—C9	14.3 (2)	C14—C13—C12—C11	−0.4 (4)
P1—O1—C14—C13	−165.8 (2)	N2—C3—C4—C5	−66.2 (2)
P1—O2—C9—C10	165.19 (17)	C12—C13—C14—O1	−179.9 (2)
P1—O2—C9—C14	−15.6 (2)	C12—C13—C14—C9	−0.1 (3)
S1—P1—O1—C14	90.71 (13)		

Symmetry codes: (i) −*x*+1, −*y*+1, −*z*+1.

### Hydrogen-bond geometry (Å, °)

**Table:**

*D*—H···*A*	*D*—H	H···*A*	*D*···*A*	*D*—H···*A*
N1—H1···S2	0.91	2.62	3.4849 (16)	160
N2—H2···S1^i^	0.91	2.60	3.2715 (16)	132
C1—H1A···O1^ii^	0.97	2.52	3.449 (2)	160

Symmetry codes: (i) −*x*+1, −*y*+1, −*z*+1; (ii) *x*, −*y*+1/2, *z*+1/2.
